# The Response Mechanism of the cbbM Carbon Sequestration Microbial Community in the Alpine Wetlands of Qinghai Lake to Changes in Precipitation

**DOI:** 10.3390/biology13121090

**Published:** 2024-12-23

**Authors:** Lin Li, Xia Wang, Yanli Yang, Siyu Wang, Kelong Chen, Ni Zhang

**Affiliations:** 1 College of Life Sciences, Qinghai Normal University, Xining 810008, China; lilinqnu@163.com (L.L.); wx_813113@163.com (X.W.); 15229584811@163.com (S.W.); 2 Qinghai Province Key Laboratory of Physical Geography and Environmental Process, College of Geographical Science, Qinghai Normal University, Xining 810008, China; 18909781391@163.com; 3 Key Laboratory of Surface Processes and Ecological Conservation on the Tibetan Plateau, Qinghai Normal University, Xining 810008, China; 4 National Positioning Observation and Research Station of Qinghai Lake Wetland Ecosystem in Qinghai, National Forestry and Grassland Administration, Haibei 812300, China; 5 Academy of Plateau Science and Sustainability, Plateau Soil Information Science Research Team, Xining 810008, China

**Keywords:** Qinghai–Tibet Plateau, precipitation pattern, climate change, carbon fixation, precipitation gradient

## Abstract

The dramatic changes in precipitation patterns on the Tibetan Plateau affected the carbon-sequestering microbial communities within wetland ecosystems, which were closely related to the responses and adaptation mechanisms of alpine wetland ecosystems to climate change. This study focused on wetland soils subjected to different precipitation gradient treatments and employed high-throughput sequencing technology to analyze the soil cbbM carbon-sequestering microbial communities. The results indicated that Proteobacteria were the dominant microbial community responsible for carbon sequestration in the Wayan Mountain wetland. A 50% increase in precipitation significantly raised the soil moisture content, while a 50% reduction and a 25% increase in precipitation notably enhanced the total soil carbon content. The 25% reduction in precipitation increased the differences in microbial community composition, whereas both the 50% increase and the 50% reduction in precipitation decreased these differences. The soil pH and temperature had the most significant impact on the carbon-sequestering microbial communities. In conclusion, changes in precipitation affect the cbbM carbon sequestration characteristics of soil microbial communities, and a moderate reduction in water input benefited carbon sequestration in wetlands.

## Simple Summary

In this paper, the mechanism of precipitation changes on the carbon sequestering microbial community of RubisCO form II gene (cbbM) in the alpine wetland of Qinghai Lake is elucidated. The dominant bacterial phylum present was Proteobacteria. A 50% increase in precipitation significantly raised the soil moisture content, while a 50% reduction and a 25% increase in precipitation notably enhanced the total soil carbon content. The 25% reduction in precipitation increased the differences in microbial community composition, whereas both the 50% increase and the 50% reduction in precipitation decreased these differences. The soil pH and temperature had the most significant impact on the carbon-sequestering microbial communities. Changes in precipitation affect the cbbM carbon sequestration characteristics of soil microbial communities, and a moderate reduction in water input may benefit carbon sequestration in wetlands.

## 1. Introduction

Wetlands are critical ecosystems in the Earth’s biogeochemical cycles, storing approximately one-third of the global soil carbon pool (450–530 Pg) [1,2]. They also play essential roles in maintaining biodiversity, conserving water resources, participating in soil carbon sequestration, and regulating the climate [3]. The alpine wetlands of the Tibetan Plateau, serving as key foundational areas for biodiversity and sensitive zones for global climate change [4,5,6], have a total area of approximately 115,600 km^2^ [7], accounting for about 30% of China’s total wetland area [8]. In recent decades, the Tibetan Plateau has experienced significant temperature increases, with annual precipitation rising at a rate of 10.2 mm per decade [9]. Moreover, in the past half-century, the frequency of precipitation during the rainy season has gradually declined, while the average intensity of single precipitation events has significantly increased [10,11], reflecting a shift in the precipitation pattern from high-frequency, low-intensity to low-frequency, high-intensity rainfall [12]. Previous studies have shown that the wetland ecosystems of the Tibetan Plateau respond markedly to climate change, with changes in precipitation often altering the carbon sequestration capabilities of wetland plants, thereby impacting the carbon source and sink functions of these ecosystems [13]. Therefore, studying the response of carbon sequestration in alpine wetlands to changes in precipitation is crucial for maintaining the high carbon storage capacity of these ecosystems.

In terrestrial ecosystems, soil serves as both a major carbon sink and a significant carbon source, and soil microorganisms are key components of the soil and play an important role in carbon sequestration and carbon mobilization within terrestrial ecosystems [14,15]. Various biogeochemical processes are closely linked to soil microorganisms [16]. They typically engage in the decomposition and transformation of organic matter through multiple metabolic pathways, aiding in the stabilization of organic carbon in soil, thereby influencing soil carbon storage and turnover [17,18]. Changes in precipitation alter water cycling and material–energy flows within wetland ecosystems, significantly impacting microbial activity in soil [19]. Shifts in precipitation patterns can further modify the structure of soil microbial communities, affecting their abundance and activity, which in turn influence the soil structure and the functions of terrestrial ecosystems [20]. Wang et al. [21] found that the methanogen community diversity in the soil of the Niao Island wetland remained relatively stable, but precipitation changes significantly influenced the microbial community composition, altering the relative abundance of certain taxa. Dong Jie [22] concluded that reduced rainfall decreased the relative abundance of Proteobacteria while increasing the relative abundance of Gram-positive bacteria, with the impacts of changing precipitation patterns differing across microbial taxa but having no significant effect on microbial diversity indices. Yang et al. [23] observed that simulated precipitation changes affected soil microbial communities and physicochemical factors, with varying impacts on microbial diversity and community composition across different soil layers under different precipitation treatments. Zhang et al. [24] reported that drastic changes in precipitation significantly reduced soil moisture and inhibited extracellular enzyme activity related to nitrogen mineralization, affecting the abundance of dominant microbes. Yang [25] found through experiments in the Songnen grassland of Jilin Province that changes in precipitation altered the bacterial diversity and structure, with soil microbial abundance influenced by various soil factors. While research on soil microorganisms in different grassland types has been extensive, studies focusing on changes in carbon-sequestering microbial communities within alpine wetland ecosystems under altered precipitation patterns remain limited.

Wayan Mountain, located at the source of the Shaliu River in Gangcha County, is part of an alpine river source wetland characterized by a cold and humid climate and rich biodiversity [26]. Numerous studies have shown that soil microbial communities in the alpine wetlands of the Tibetan Plateau exhibit considerable fluctuations in response to environmental changes, indicating a relatively fragile nature [27]. Currently, more in-depth research is needed on the effects of changes in precipitation on the structure and diversity of carbon-sequestering microbial communities in alpine wetlands, as well as their impacts on the structure and function of wetland ecosystems. This study focuses on the Wayan Mountain wetland and employs a precipitation manipulation system with varying levels of increased and decreased rainfall. Using high-throughput sequencing technology, we aim to characterize the microbial community associated with the cbbM functional gene and investigate the impact of precipitation changes on the diversity and structure of carbon-sequestering microbial communities. By analyzing the interactions between carbon-sequestering microorganisms and environmental factors under different precipitation conditions, this study further explores the mechanisms that regulate carbon sources and sinks in alpine wetlands.

## 2. Materials and Methods

### 2.1. Overview of the Study Area

The study area is located at the Wayan Mountain Field Observation Station (37°44′34″ N, 100°05′41″ E, elevation 3720–3850 m) in Gangcha County of the Haibei Tibetan Autonomous Prefecture on the northern shore of Qinghai Lake, approximately 52 km northwest of Gangcha County. This area is classified as a river source wetland. The long-term average temperature is −3.31 °C, with daily average temperatures ranging between 11.87 °C and 19.73 °C, indicative of a plateau continental climate. Precipitation is abundant at this site and predominantly occurs from June to August, with an annual total of 420.37 mm. The dominant vegetation at the observation station is Kobresia humilis, with Carex tristachya, Lobularia maritima, and Potentilla anserina species serving as associated plants. The soil layer of Wayan Mountain is often in a water-logged state, the main soil type is mollic gleysols, and the layer beneath the surface soil consists of alluvial deposits [28]. Key characteristics of the observation station include ample sunlight and a marked diurnal temperature variation.

### 2.2. Sample Plot Design and Soil Sample Collection

The experimental plots were established on a flat meadow area with uniform vegetation and a large surface area in August 2018. Each plot measured 30 m × 30 m and was arranged in a 3-by-3 grid pattern with 3 m × 5 m buffer zones between plots. Metal sheets were inserted between plots to prevent surface runoff, and control and four different precipitation treatment groups were established. Reduced precipitation treatments of 25% (Jb) and 50% (Ja) were achieved using concave transparent rain exclusion panels covering 25% and 50% of the plot area, respectively. Increased precipitation treatments of 25% (Zb) and 50% (Za) were implemented by collecting rainwater with an 11° inclined structure that funneled water into barrels, from which the collected rainwater was uniformly sprayed onto the plots [29,30]. In June 2020, soil samples were collected from a depth of 0–10 cm within each plot using a 4.5 cm diameter soil auger and following a five-point sampling method. The collected samples from the same layer were mixed and passed through a 2 mm soil sieve for homogenization. Portions of the soil samples were placed in 10 mL EP tubes and stored in liquid nitrogen for subsequent DNA extraction, while the remaining samples were sealed in plastic bags and transported to the laboratory for physicochemical analysis.

### 2.3. Determination of Soil Physical and Chemical Properties

Soil moisture at a depth of 0–10 cm was monitored using a TDR-300 (produced by Spectrum Technologies in Plainfield, IL, USA). Soil temperature within the same depth range was measured using an LI-8100 instrument (manufactured by LI-COR in Lincoln, NE, USA). For soil pH measurement, soil was mixed with water in a 1:2.5 ratio, and then the pH was determined using a pH meter (model FE20-FiveEasy pH, from MeLler Toledo in Gießen, Germany). Total carbon (TC) and total nitrogen (TN) content were analyzed using an elemental analyzer system (Vario EL III, Elemental Analysis System GmbH, Langenselbold, Germany) [31].

### 2.4. Soil DNA Extraction and Library Construction

The total DNA from soil samples was extracted using the PowerSoil DNA Isolation Kit (Mio-bio, Carlsbad, CA, USA). Qualified DNA samples were selected for subsequent PCR amplification using cbbM-specific primers, including the forward primer (5′-TTCTGGCTGGGBGGHGAYTTYATYAARAAYGACGA-3′) and the reverse primer (5′-CCGTGRCCRGCVCGRTGGTARTG-3′). Sequencing was performed on an Illumina MiSeq platform [32].

### 2.5. Statistical Analysis

Functional groups of microorganisms were predicted using FAPROTAX [33]. Statistical analyses and visualizations, including *p*-value calculations and plotting, were performed using R version 4.1.2 [31].

## 3. Results

### 3.1. Community Diversity of cbbM Carbon Sequestration Microorganisms Under Different Precipitation Treatments

**Figure 1:**
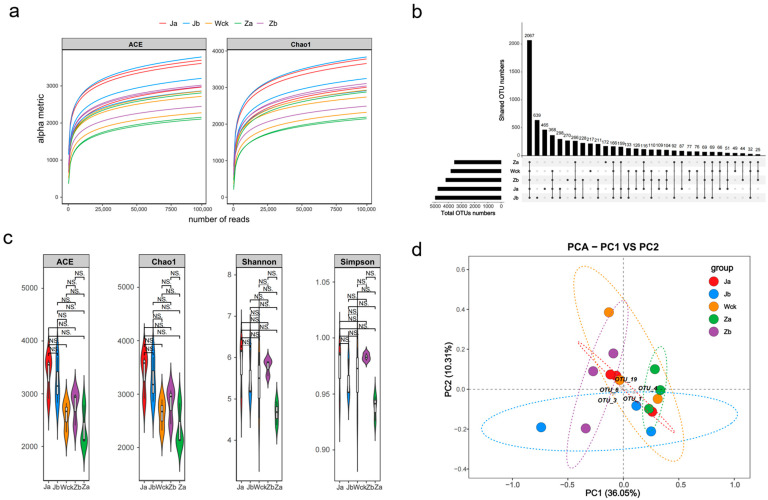
Illumina sequencing results and carbon sequestration microbial community diversity; (**a**) sample dilution curve; (**b**) OTU distribution map; (**c**) cbbM microbial alpha diversity index; (**d**) cbbM microbial principal component analysis. NS indicates *p* > 0.05.

The rarefaction curves of the soil samples demonstrated a plateauing trend, indicating sufficient sequencing depth and a comprehensive reflection of species diversity within the samples. A total of 6961 operational taxonomic units (OTUs) were obtained from the cbbM microbial communities in the soil samples. Most of the OTU counts remained consistent across samples (Figure 1b), suggesting a high degree of similarity among coexisting species within these samples. No statistically significant differences in the alpha diversity indices of the cbbM communities were observed under different precipitation treatments (Figure 1c, *p* > 0.05). The richness indices (ACE, Chao1) of the cbbM communities followed the order Ja > Jb > Za > Wck > Zb, while the diversity indices (Shannon, Simpson indices) were ranked as Ja > Zb > Wck > Jb > Za. Principal component analysis (PCA) of the OTU composition, after noise and redundancy removal for dimensionality reduction, showed that the first and second principal components contributed 36.05% and 10.31% of the variance, respectively, with a cumulative contribution of 46.36% (Figure 1d). Overall, the cbbM community structures under different precipitation treatments were relatively similar, with the soil heterogeneity in Za and Ja being lower compared to the control (Wck). However, Jb exhibited the highest heterogeneity, indicating a more pronounced difference in the composition of carbon-sequestering microbial communities.

### 3.2. Composition of cbbM Carbon Sequestration Microbial Communities Under Different Precipitation Treatments

**Figure 2:**
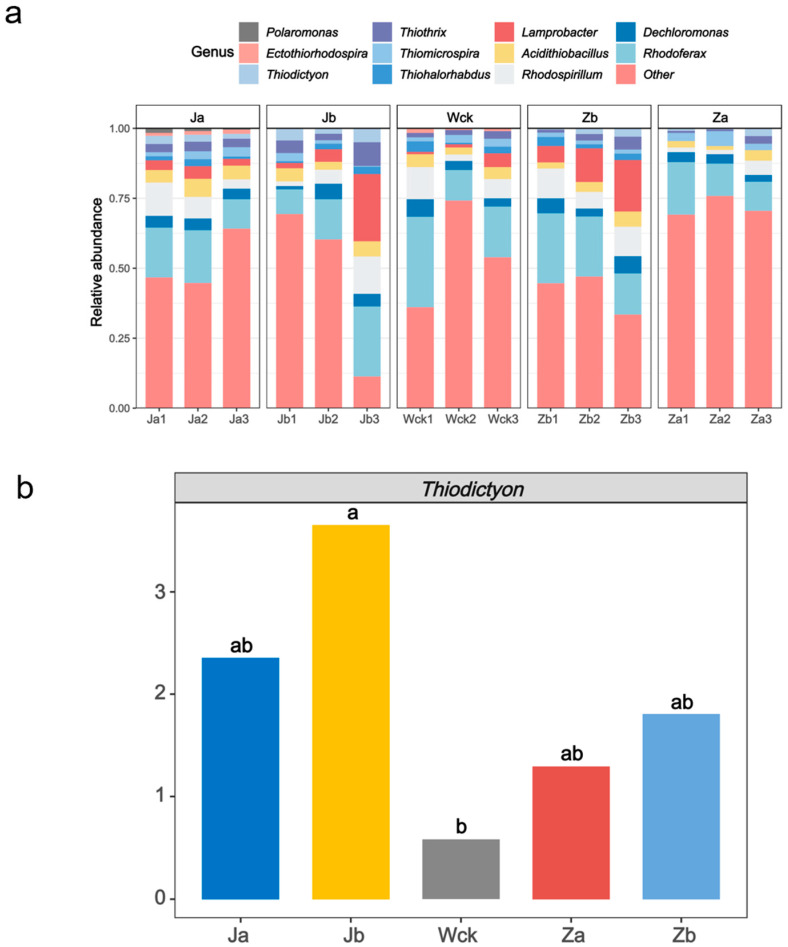
Microbial community composition; (**a**) community composition of cbbM carbon sequestration microorganisms; (**b**) genera-level difference in *Thiodictyon*. ab indicates significance, the same letter indicates no significant difference between groups (*p* > 0.05), and different letters indicate a significant difference between groups (*p* < 0.05).

At the phylum level, Proteobacteria were the dominant bacterial group, and an analysis of the cbbM communities at the genus level (Figure 2a) showed that the relative abundance of unclassified taxa in the soil samples treated with Ja, Jb, Wck, Zb, and Za was 51.59%, 46.81%, 54.49%, 41.59%, and 71.59%, respectively. Among the identified genera, dominant taxa included *Thiodictyon*, *Rhodoferax*, *Dechloromonas*, *Rhodospirillum*, *Acidithiobacillus*, *Lamprobacter*, *Thiohalorhabdus*, *Thiomicrospira*, *Thiothrix*, *Ectothiorhodospira*, and *Polaromonas*. *Rhodoferax* was consistently observed as the genus with the highest relative abundance in the soil microbial communities across all precipitation treatments, with respective values of 15.65%, 16.09%, 20.43%, 20.29%, and 13.62%. The abundance of *Thiodictyon* varied among the different precipitation treatments. Variance analysis conducted on *Thiodictyon* (Figure 2b) demonstrated significant differences in community composition between the Jb and Wck treatments.

### 3.3. Functional Groups of the cbbM Carbon Sequestration Microbial Community Under Different Precipitation Treatments

**Figure 3:**
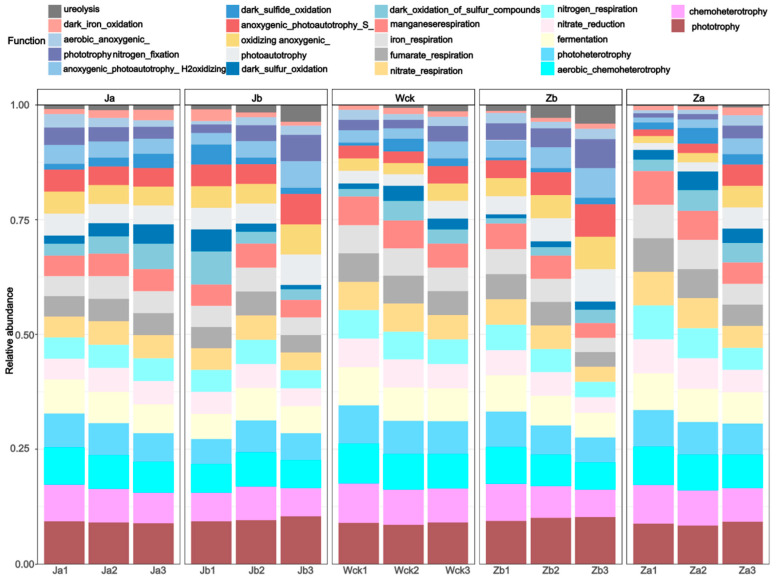
Main functional groups of cbbM carbon sequestration microorganisms.

**Figure 4:**
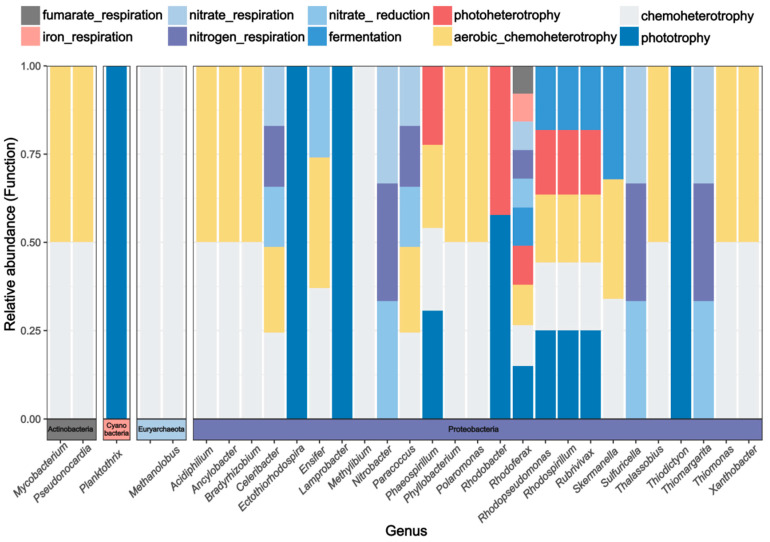
The main functional groups of the C cycle and the corresponding generic-level microflora.

The FAPROTAX functional annotation results of the soil cbbM communities under different precipitation treatments (Figure 3) indicated that carbon-fixing microorganisms were categorized into 22 major ecological functional groups (relative abundance > 0.1%). The primary microbial functions associated with cbbM included phototrophy, chemoheterotrophy, aerobic_chemoheterotrophy, photoheterotrophy, fermentation, nitrate_reduction, nitrogen_respiration, nitrate_respiration, fumarate_respiration, and iron_respiration. An analysis of the major soil functional groups and their genus-level microbial communities (Figure 4) revealed that soil cbbM carbon-fixing microorganisms were distributed among 30 genus-level groups across four phyla, with 25 of these genera belonging to Proteobacteria, indicating that Proteobacteria dominated the carbon-fixing microbial community. Most bacteria exhibited predominant functional groups of chemoheterotrophy and aerobic chemoheterotrophy, suggesting that they gain energy for growth and reproduction through the oxidative decomposition of organic matter. Additionally, some anaerobic respiration functional groups, such as iron_respiration and nitrogen_respiration, were present, aiding the microbial community’s adaptation to hypoxic soil environments.

### 3.4. Correlations Between the cbbM Carbon Sequestration Microbial Community and Soil Environmental Factors Under Different Precipitation Treatments

**Figure 5:**
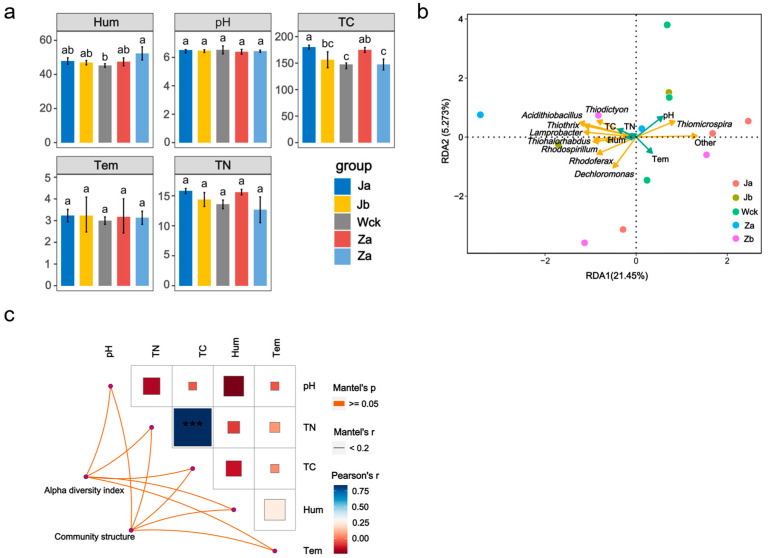
Correlation between soil environmental factors and carbon-sequestering microorganisms; (**a**) changes in soil physical and chemical factors under different precipitation treatments; (**b**) redundancy analysis of environmental factors and genus-level microflora (Top 10); (**c**) correlation network diagram between carbon-sequestering microbial community characteristics and environmental factors. abc indicates significance, the same letter indicates no significant difference between groups (*p* > 0.05), and different letters indicate a significant difference between groups (*p* < 0.05); *** indicates *p* < 0.001.

Soil physicochemical factors were significantly influenced by changes in precipitation (Figure 5a). The study found that the soil moisture content under the Za treatment was significantly higher than that of the other precipitation treatments, while the total carbon and total nitrogen values were lower. Treatments Zb and Ja increased the total carbon content in the soil. There were no significant differences in the soil pH, temperature, and total nitrogen content in response to precipitation changes. The α-diversity index and community structure of carbon-fixing microorganisms did not show a significant positive correlation with the soil physicochemical factors, although there was a relatively strong correlation between the total nitrogen and total carbon (Figure 5b). Redundancy analysis of the top 10 dominant microbial groups in terms of relative abundance and soil physicochemical factors (Figure 5c) concluded that different factors affected the microorganisms differently, with the pH and temperature being the main soil physicochemical factors influencing the community of carbon-fixing microorganisms.

### 3.5. Correlation of Soil cbbM Community Characteristics with Environmental Variables Under Different Precipitation Treatments

**Figure 6:**
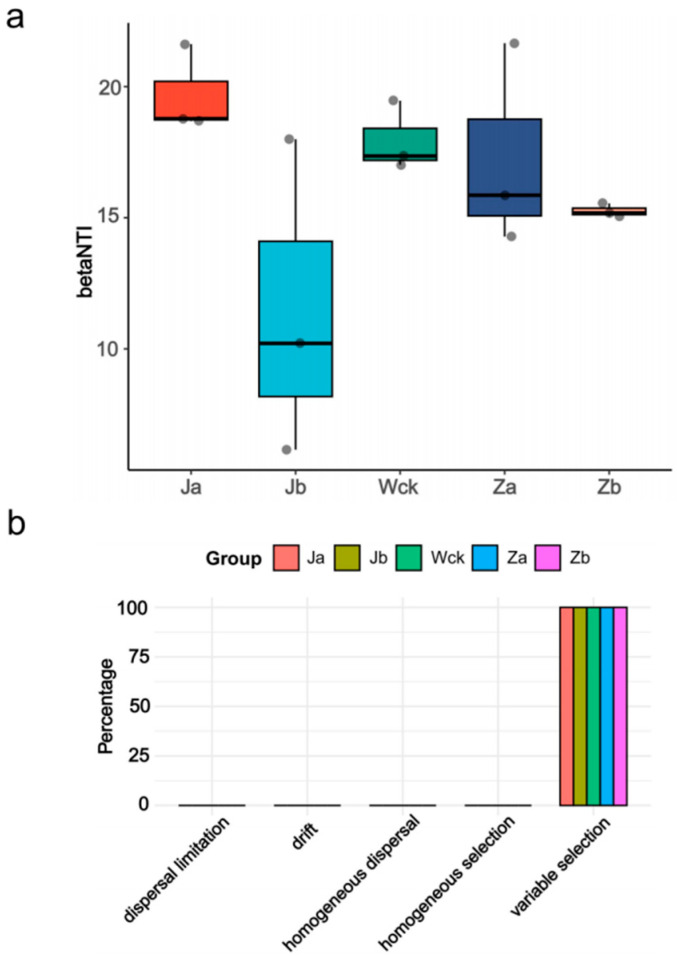
(**a**) betaNTI index of different groups; (**b**) distribution of community construction process in different groups.

Deterministic processes drive the community assembly of nirStype denitrifiers. The calculation of βNTI values for community assembly under different precipitation treatments shows that deterministic processes dominate (|βNTI| > 2) at this regional scale (Figure 6a). Further calculation of RCbray distinguishes the relative impacts of dispersal limitation, drift, homogeneous dispersal, and selection on community dynamics (Figure 6b). The results indicate that the composition of nirS-type denitrifying bacterial communities in the Qinghai Lake wetland was solely influenced by deterministic processes. Under all precipitation treatments, heterogeneous selection played a 100% role.

## 4. Discussion

### 4.1. Effects of Different Precipitation Treatments on cbbM Carbon Sequestration Microbial Community Diversity

Soil water availability is significantly positively correlated with rainfall, and changes in precipitation can directly alter soil moisture, thereby affecting the diversity of soil cbbM carbon-fixing microbial communities [34]. In their study, Lian et al. observed that variations in precipitation significantly impacted plant and soil microbial diversity in desert steppes [35]. Through a meta-analysis, Ren et al. concluded that reduced precipitation significantly decreased soil bacterial abundance [36]. However, this experiment found that changes in precipitation did not significantly affect microbial community diversity. Similar conclusions have been drawn by other scholars; for instance, Sun Xin et al. found that long-term increased precipitation in semi-arid grasslands near Stanford University did not significantly alter soil microbial community diversity or species composition [37]. Liu et al. simulated South Subtropical environments and found no significant effect of precipitation treatments on microbial community structure [38]. Additionally, Tian Rui et al. observed in their study of the semi-arid Loess Plateau region in Gansu that reduced precipitation did not significantly affect soil bacterial community composition and diversity [39]. Kwon et al. found in their experiment in northeastern Siberia that soil bacterial and functional α-diversity did not differ significantly following drainage [40]. Moreover, this study found a reduced difference in carbon-fixing microbial communities under the Ja and Za treatment conditions, which might be attributed to the altered vegetation structure caused by substantial precipitation increases or decreases, subsequently suppressing soil microbial activity [41,42].

The diversity of carbon-fixing microbial communities on the Qinghai–Tibet Plateau is closely related to environmental factors, with soil moisture and pH considered key determinants of the composition and function of soil microbial communities [43,44]. This study showed that Za treatment significantly increased the soil moisture content, while the Zb and Ja treatments significantly raised the total soil carbon levels. No significant differences were observed in the soil pH, temperature, or total nitrogen content due to changes in precipitation. However, further analysis revealed that the soil pH and temperature differentially affected the top 10 most abundant microbial taxa. Hu et al. demonstrated that soil moisture could influence microbial diversity to some extent [45]. Similarly, Wang et al. found that the pH can impact microbial communities by modulating the carbon and nitrogen content [46]. The lack of significant differences in the soil pH, temperature, and total nitrogen content in response to precipitation changes may be attributed to large variations in precipitation across temporal and spatial scales [47].

### 4.2. Effects of Different Precipitation Treatments on cbbM Carbon Sequestration Microbial Community Structure

Long-term changes in precipitation primarily influence the living environment of microorganisms by altering the soil moisture, pH, temperature, and other physicochemical properties, as well as vegetation diversity, thereby directly impacting their community structure [48]. Variations in the abundance of carbon-fixing microbial communities are often used to indirectly reflect carbon sequestration potential [49]. Changes in rainfall and its seasonality can affect the availability of water for plants and microorganisms, thereby influencing the abundance of soil microbial communities [50]. The study by Wang et al. demonstrated that changes in precipitation significantly affected microbial relative abundance [51]. Similarly, Yang Jie et al. emphasized that soil moisture has a significant impact on the soil bacterial community structure and diversity index [52]. This study found that reducing rainfall by 50% and increasing it by 25% led to an increase in the richness of cbbM communities. Wang Xia reported that variations in rainfall in the temperate grasslands around Qinghai Lake did not significantly alter soil microbial richness [53]. Likewise, the study by Wang et al. showed that precipitation changes influenced the structure and composition of soil microbial communities in the Changbai Mountain primary forest [54]. Myeong et al. conducted a drainage experiment in northeastern Siberia and found that microbial richness did not differ significantly across all soil depths under the drainage treatment [55]. Genus-level analysis of cbbM communities revealed that *Rhodoferax* exhibited the highest relative abundance across all treatments, while *Thiobacillus* demonstrated variability under different precipitation treatments, with significant differences observed between Jb and Wck treatments. This suggests that changes in the soil moisture content have a notable impact on the presence of soil microorganisms [56]. Taş et al. found in their study that microtopographic features of polygonal tundra that regulate the soil water distribution affect the structure and function of soil microbial communities [57]. This study also indicated that Proteobacteria were dominant in the carbon-fixing microbial communities within the plots, consistent with the findings of Zhang et al. in their study on carbon-fixing microorganisms in three wetland types around Qinghai Lake [58]. Proteobacteria play a critical role in the global carbon cycle and have significant impacts on soil nutrient cycling and broader biogeochemical processes [59]. Ma et al. identified Proteobacteria as key carbon-fixing microorganisms across different wetland types [60]. Liao et al. investigated the response of carbon-fixing microbial communities in coastal marsh wetland soils to changes in plant communities and found that Proteobacteria were the most important carbon-fixing group [61]. These findings align with the results of this study. Correlation analysis of the physicochemical factors and soil carbon-fixing microorganisms revealed that changes in the community structure were primarily driven by shifts in the total soil carbon and nitrogen content. Additionally, Wang et al. highlighted that changes in soil carbon fractions are key factors influencing the structure of carbon-fixing microbial communities in wetland soils [62].

## 5. Conclusions

This study compared the characteristics of cbbM carbon-fixing microbial communities and their correlation with soil environmental factors under different precipitation treatments in the river source wetlands of Qinghai Lake. It was found that there were no significant differences in the α-diversity of carbon-fixing microorganisms, while the 50% increase and 50% decrease in precipitation treatments reduced the differences in microbial community composition. The dominant species composition of soil cbbM carbon-fixing microbial communities was similar under different precipitation treatments, with Proteobacteria as the dominant phylum and *Rhodoferax* as the genus with the highest relative abundance across all rainfall increase and decrease treatments. Among the environmental factors, the soil pH and temperature had the most significant impact on the carbon-fixing microbial communities. Different precipitation treatments in the wetlands led to changes in environmental factors; a 50% increase in precipitation significantly enhanced the soil moisture content, while a 50% reduction and a 25% increase in precipitation significantly elevated the total soil carbon content. This study aimed to provide scientific evidence and reference for soil carbon fixation and ecological protection in alpine wetland ecosystems.

## Data Availability

The raw data have been uploaded to NCBI, and its BioProject is PRJNA1188060.
